# Galangin/β-Cyclodextrin Inclusion Complex as a Drug-Delivery System for Improved Solubility and Biocompatibility in Breast Cancer Treatment

**DOI:** 10.3390/molecules27144521

**Published:** 2022-07-15

**Authors:** Zainab S. Abbas, Ghassan M. Sulaiman, Majid S. Jabir, Salman A. A. Mohammed, Riaz A. Khan, Hamdoon A. Mohammed, Amal Al-Subaiyel

**Affiliations:** 1 Division of Biotechnology, Department of Applied Science, University of Technology, Baghdad 10066, Iraq; zainabsalim21@gmail.com (Z.S.A.); 100131@uotechnology.edu.iq (M.S.J.); 2 Department of Pharmacology and Toxicology, College of Pharmacy, Qassim University, Qassim 51452, Saudi Arabia; m.azmi@qu.edu.sa; 3 Department of Medicinal Chemistry and Pharmacognosy, College of Pharmacy, Qassim University, Qassim 51452, Saudi Arabia; ri.khan@qu.edu.sa (R.A.K.); ham.mohammed@qu.edu.sa (H.A.M.); 4 Department of Pharmacognosy and Medicinal Plants, Faculty of Pharmacy, Al-Azhar University, Cairo 11371, Egypt; 5 Department of Pharmaceutics, College of Pharmacy, Qassim University, Qassim 51452, Saudi Arabia

**Keywords:** galangin, β-cyclodextrin, drug delivery, biocompatibility, cytotoxicity, caspase-3 pathway, MCF-7 cells, REF cells

## Abstract

The purpose of this study was to evaluate the potential of a newly modified cyclodextrin derivative, water-soluble β-cyclodextrin–epichlorohydrin (β-CD), as an effective drug carrier to enhance the poor solubility and bioavailability of galangin (GAL), a poorly water-soluble model drug. In this regard, inclusion complexes of GAL/β-CDP were prepared. UV-VIS spectrophotometry, Fourier-transform infrared spectroscopy (FTIR), X-ray crystallography (XRD), zeta potential analysis, particle size analysis, field emission scanning electron microscopy (FESEM), and transmission electron microscopy (TEM) were applied to characterize the synthesized GAL/β-CD. Michigan Cancer Foundation-7 (MCF-7; human breast cancer cells) and rat embryo fibroblast (REF; normal cells) were employed to examine the in vitro cytotoxic effects of GAL/β-CD using various parameters. The dye-based tests of MTT and crystal violet clearly exhibited that GAL/β-CD-treated cells had a reduced proliferation rate, an influence that was not found in the normal cell line. The cells’ death was found to follow apoptotic mechanisms, as revealed by the dye-based test of acridine orange/ethidium bromide (AO/EtBr), with the involvement of the mitochondria via caspase-3-mediated events, as manifested by the Rh 123 test. We also included a mouse model to examine possible in vivo toxic effects of GAL/β-CD. It appears that the inclusion complex does not have a significant influence on normal cells, as indicated by serum levels of kidney and liver enzymatic markers, as well as thymic and splenic mass indices. A similar conclusion was reached on the histological level, as manifested by the absence of pathological alterations in the liver, kidney, thymus, spleen, heart, and lung.

## 1. Introduction

While the global mortality rate is attributed to various factors, cancer plays a leading role, especially in the developing countries where it is rising as a serious issue [1,2]. The estimates from 2020 stated that 19.3 million additional cases of cancer are expected to be reported globally (18.1 million with the exception of the National Merit Scholarship Corporation (NMSC), excluding skin cancers) with 10 million cancer-caused deaths (9.9 million with the exception of the National Merit Scholarship Corporation (NMSC), excluding skin cancers) [3]. Internal (such as hormones, inherent mutations, and immune conditions) as well as external (including cigarettes, food, infectious organisms, and radiation) factors might cause cancer [4].

Breast cancer (BC) remains the second greatest cause of mortality globally in females. According to Cancer Statistics 2020, BC accounts for 30% of all female cancers, with 276,480 new cases and over 42,000 expected fatalities [5]. A breast cancer gene 1 (BRCA1) or BRCA2 gene mutation is thought to be responsible for 2% to 3% of all cases of BC [6]. In several countries, limited access to affordable, high-quality cancer surgery, pathology tests, essential cancer medicines, and radiotherapy are all factors that influence poorer survival in many countries [7]. In addition, treatment interventions such as chemotherapy and radiotherapy can have serious side effects, while patients can still acquire resistance to them [8].

Flavonoids are among the most diverse classes of bioactive molecules, with approximately 9000 different chemicals [9]. Flavonoids form the most diverse class of polyphenols, with a wide range of functional and structural properties [10]. The name “flavonoid” refers to phenyl-substituted propylbenzene compounds and flavonolignans formed from phenyl-substituted propylbenzene compounds combined with C6-C3 flavonoid substrates having a C15 backbone [11]. Flavonoids have been divided into ten categories based on their chemical structures, with flavones, flavanones, anthocyanidins, flavonols, isoflavones, and catechins being the most common in the human diet. Many of these flavonoids have been shown to have anti-tumor properties in both in vivo and in vitro studies [12].

Galangin (3,5,7-trihydroxy-2-phenylchromen-4-one; 3,5,7-trihydroxyflavone) is a natural flavonoid found in *Alpinia officinarum* Hance (Zingiberaceae) and honey [13,14]. Galangin has already been proven to possess a wide spectrum of medicinal benefits, exerting anti-inflammatory, antiarthritic, hepatoprotective, antidiabetic, and antibacterial properties [13]. Galangin also possesses antioxidant, free radical scavenging, hypolipidemic, antitumor, enzyme regulative, antifungal, and anti-Alzheimer’s disease effects [15,16]. Galangin is a non-toxic chemical for humans, but it is toxic to cancer cells, making it a potential anticancer medication [17]. In recent investigations, galangin has been shown to trigger programmed cell death and enhance autophagy [16].

Cyclodextrins (CDs) are cyclic oligosaccharides made from starch by enzymatic hydrolysis. They are a kind of cyclic oligosaccharide having surface hydrophilic and interior hydrophobic qualities that have been widely employed in cancer therapy. Nanoparticle-based treatment, gene therapy, cell therapy, immunotherapy, and chemotherapeutics have all used cyclodextrin [18]. CDs are non-toxic and have a hollow structure that may catch a specific organic molecule, making them excellent as adsorbent materials [19].

Nanotechnology is the study of creating and utilizing materials having unique characteristics at nanoscale levels, allowing for innovative uses [20]. It has been used in the creation of nanomaterials. Delivery of drugs, gene therapy, monitoring and diagnostics, drug transport, biomarker identification, targeted therapy, and biomolecular applications are just a few of the intriguing uses of nanotechnology in diagnostics and therapy [21].

Engineered materials with a size between 1 nm and 100 nm in at least one dimension are referred to as nanoparticles or nanomaterials. They are divided into two groups: organic and inorganic [22]. Because of their physicochemical and biological features, nanoparticles offer a wide range of biological uses, including biosensing, biological separation, molecular imaging, and anticancer treatment. Nanoparticles of various types have different features that can be utilized to target cancer cells [23]. These include cell/tissue permeability, specific biodistribution, circulatory half-life, aggregation at target sites, drug-loading capability, and low toxic effect [24,25]. The present study attempts to synthesize galangin within β-cyclodextrin inclusion complex as a drug-delivery system with improved solubility and biocompatibility for BC treatment. The efficiency of the complex was assessed on human BC cells (MCF-7). We also examined to what extent it is biocompatible with human serum and red blood cells. Additionally, we included a mouse model to examine possible in vivo toxic effects of GAL/β-CD in terms of serum levels of liver and kidney function markers as well as possible histopathological changes in a number of organs.

## 2. Materials and Methods

### 2.1. Materials and Reagents

Galangin (GAL; purity ~ 98%) and β-cyclodextrin (β-CD) were purchased from Biotech Co., Changchun, China. Ethanol was purchased from Lobacheme, Mumbai, India. Acridine orange, ethidium bromide, trypsin-EDTA, dimethyl sulfoxide (DMSO), 3-(4,5-dimethylthiazal-z-yl)-2,5-diphenylterazolium (MTT), fluorescein isothyocyanate (FITC), rhodamine (Rh 123), crystal violate stain, and fetal bovine serum (FBS) were ordered from Sigma Chemical Co. (St. Louis, MO, USA). Dulbecco’s Modified Eagle Medium (DMEM) was supplied by Euro Clone (Milan, Italy). The fluorescein caspase-3 staining package was supplied by Thermo Fisher Scientific, Rockford, IL, USA. Antibiotics (penicillin and streptomycin) were supplied by Biosource International, Nivelles, Belgium. All the other chemicals and reagents used in this investigation were employed in their analytical grades.

### 2.2. Cell Line Cultures

Both the MCF-7 and REF cell lines were generously supplied by the Iraqi Center for Cancer and Medical Genetic Researches (ICCMGR), Mustansiriyah University, Baghdad, Iraq. MCF-7 or REF cultures were maintained in Falcon 25 cm^2^ tissue culture flasks (Falcon, Washington, WA, USA) containing DMEM with supplements of 10% FBS, 2 mM L-glutamine, and 20 mM HEPES. The culture was incubated at a CO_2_ concentration of 5% and a temperature of 37 °C.

### 2.3. Preparation of Galangin and β-Cyclodextrin Inclusion Complexes

Galangin and β-cyclodextrin inclusion complexes (GAL/β-CD) were synthesized by using the nanoparticipation technique [26] with some modifications. Briefly, 0.238 g of GAL was resolved in 25 mL of ethanol and sonicated for 15 min. Then, 1.59 g of β-CD was resolved in 50 mL of deionized water (D.I.W), i.e., 31.8 mg/mL^−1^. The GAL solution was added to the β-cyclodextrin solution in a drop-wise manner and mixed and stirred (1000 rpm) at room temperature for 30 min. After that, the emulsion was transferred to 20 mL of D.I.W. and stirred for 12 h at room temperature to simplify diffusion and ensure total vaporization of the organic solvent. After precipitation and filtration, the supernatant containing the water-soluble GAL/β-CD complex was recovered by freeze-drying. Then, the GAL/β-CD complex was re-suspended in 20 mL of D.I.W for further use. The percentage of encapsulated galangin in β-CD complex was calculated using the following equation:

(1) $$\mathrm{Encapsulation}\;\left(\%\right)=\frac{\mathrm{Weight}\;\mathrm{of}\;\mathrm{galangin}\;\mathrm{in}\;\mathrm{NPs}}{\mathrm{Weight}\;\mathrm{of}\;\mathrm{initial}\;\mathrm{drug}}\times 100$$

### 2.4. Release Profile of GAL/β-CD

The release profile of the modified GAL/β-CD inclusion complex was performed as described in the previous study by Mora et al. [27]. A weight of 3 mg of galangin and 3 mg of GAL/β-CD were suspended in 3 mL of phosphate buffer saline (PBS) solution of either physiological pH (7.4) or acidic pH 5.04 and incubated at 37 °C for different periods of time (5, 10, 15, 30, 60, 120, 180, 240, 300, 360, 420, 480, 540, and 600 min). At each time point, the absorbance was measured for released galangin (free galangin at pH 7.4) or released galangin in β-CD inclusion at pH 7.4 and pH 5.04 by using a UV/VIS spectrophotometer (360 nm). Then, the percentage of cumulative release of samples at each time point was detected by utilizing the following formula:

(2) $$\;\mathrm{Camulative}\;\mathrm{Release}\;\%=\frac{\mathrm{Wr}}{\mathrm{Wt}}\times 100$$

where Wr is galangin weight release, and Wt is galangin weight loading.

### 2.5. Characterization of GAL/β-CD

As an initial step, the Shimadzu Europe—UV-1650PC Spectrophotometer (Tokyo, Japan) was utilized to conduct the UV–vis spectral characterization of galangin and GAL/β-CD by continuous scanning (200–500 nm). The crystals formed by the synthesized complexes were examined by applying X-ray diffractometric analysis (XRD-6000, ADX-2700, USA) under the conditions of 30 mA current and 40 kV voltage. Diffraction patterns were analyzed for particles subjected to a Cu Kα incident beam at λ = 1.540 A° and 2θ = 5–40°. An FTIR 8400S device (Tensor 27, Amsterdam, The Netherlands) was utilized to conduct the Fourier spectroscopic analysis by applying the attenuated total reflection mode under conditions of a 4000–400 cm^−1^ spectral range and 4 cm^−1^ resolution. A Brookhaven Zeta PALS device supplied by Milton Keynes, UK, was employed for the characterization of additional properties of the synthesized nanoparticles (i.e., zeta potential and particle size). The produced complex was also characterized in terms of morphology by using FESEM analysis with MIRA 3 TESCAN (Brno, Czech Republic). This characterization, in addition to the distribution pattern of the complex, was confirmed by TEM examination (Zwiss, Oberkochen, Germany) working at 400 kV.

### 2.6. Serum Stability Study

The physical stability of pure galangin and GAL/β-CD was investigated in a physiologically appropriate medium of FBS (10%), of which 10 mL were used for the incubation of unconjugated galangin and GAL/β-CD (1 mL each). The combinations were kept at 37 °C and lightly stirred (200 rpm) to replicate blood circulation conditions. To determine the values of polydispersity index (PDI) and particle size (PS), samples were diluted with distilled water (1:50 *v*/*v*) following predetermined time intervals of 2, 4, and 6 h [28].

### 2.7. Sampling and Preparation of Human Blood

Samples of human fresh blood were obtained, based on methods described elsewhere [29] as well as standard guidelines of the NIH and FDA and the ethical principles of the Helsinki Declaration, and placed into heparin-coated tubes [29]. The study was permitted following the regulations of the medical city in Baghdad, Iraq, while an official approval under ref. No. AS 921/5/10/2020 was obtained from the ethical committee, University of Technology, Baghdad.

### 2.8. Blood Compatibility Assay

A mixture of whole blood (200 µL), normal saline (1600 µL), and galangin or GAL/β-CD (200 µL; concentration range of 20–640 µg mL^−1^) was prepared. Samples of positive control (D.W; 100% lysis) and negative control (normal saline; 0% lysis) were also examined. All samples were then subjected to incubation in a water bath (1 h, 37 °C). The samples were then centrifuged for 5 min at 700 rpm. The absorbance of the supernatant for each sample was measured using UV–vis spectrophotometry at 541 nm [30]. The percent of hemolysis was calculated utilizing the following equation:

(3) $$\mathrm{Hemolysis}\;\%=\left(\frac{{\mathrm{OD}}_{s}-{\;\mathrm{OD}}_{n}\;}{{\mathrm{OD}}_{p}-{\;\mathrm{OD}}_{n}}\right)\times 100\;$$

where OD_s_ is the optical density for the sample under test, OD_n_ is that for negative control, and OD_p_ is that for positive control.

To obtain photographs, one drop of each blood sample (controls and treated) was smeared on a slide and left to dry at an ambient temperature. Smears were then stained with Giemsa and photographed by a light microscope.

### 2.9. Cytotoxic Impacts of the Inclusion Complex

The tested cell lines (200 μL, 1 × 10^5^ cells mL^−e^) were suspended and cultured in 96-well flat-bottom plates (Falcon, Lincoln Park, NJ, USA). Following 48 h maintenance in the exponential growth phase, the cells were treated for 24 h with free galangin and GAL/β-CD. MTT in PBS (100 µL) was then used to label the well-maintained cells for 10–15 min at 37 °C. Tap water was used to wash excess dye, whereas treatment with 50 µL DMSO for 10 min was applied to dissolve air bubbles. The absorbance of cell cultures was measured by using a microplate reader (ELx 800, Bio-Tek Instruments Inc., Winooski, VT, USA) at 492 nm [29]. The calculation of the rate of inhibition of cell growth was performed by applying the following equation:

(4) $$\mathrm{Inhibition}\;\mathrm{rate}\;\left(\%\right)=\frac{\mathrm{Abc}\;-\;\mathrm{Abs}}{\mathrm{Abc}}$$

where Abc and Abs refer to the OD values for the control and tested samples, respectively.

### 2.10. Staining of Cells with Crystal Violet 

For staining with crystal violet, similar conditions to those of the MTT test were applied. In brief, crystal violet dye (50 µL) was applied to replace the medium at the end of the recovery time. Following 10 min of incubation, tap water was gently applied thrice to the cells to remove excess stain, followed by rinsing with distilled water and air-drying. Examination of the morphological features of cells was achieved by means of a phase-contrast inverted microscope (magnification power: 40×) [31].

### 2.11. Acridine Orange–Ethidium Bromide (AO/EtBr) Dual Staining

The AO/EtBr double staining assay was employed to detect cell death and nuclear morphologic aspects. Cultures of MCF-7 or REF cells were grown in 96-well plates. After achieving confluence, the cells were exposed to the IC_50_ concentrations of galangin and GAL/β-CD in serum-free media and incubated at 37 °C and 5% CO_2_ for 24 h. Control cells were treated with serum-free media only. Following the incubation, the medium was removed and washed with PBS; then, 50 µL of acridine orange–ethidium bromide stain mixture was added to the cells, which were incubated at 37 °C for 15 min. After staining, the plate was washed with PBS. The cells were observed immediately under the inverted fluorescence microscope at 40× magnification [29]. 

### 2.12. Mitochondrial Activity

Rhodamine (Rh123) is a fluorescent dye that was applied to provide further confirmation for the impacts of the examined compounds on the activity of the mitochondria in MCF-7 cells. Briefly, MCF-7 and REF cells cultured in 96-well plates (104 cells mL^−1^) were subjected to treatment with IC_50_ concentrations of pure galangin and GAL/β-CD inclusion complex, followed by staining with Rh123 (5 M, 2 h, 37 °C). Detachment of cells was then achieved by applying trypsin–EDTA (0.2 mL of 5%), followed by centrifugation (300 rpm for 5 min). Cells were re-suspended in FACS buffer and examined by a flow cytometer, from which histogram plots were extracted. Rh123 was utilized for the examination of the potential of the mitochondrial membrane both pre-and post-treatment with the compounds under investigation [32].

### 2.13. ELISA Assay

Cultures of MCF-7 and REF cells (5 × 10^5^ cell mL^−1^) were prepared in 96-well plates. Cells were then incubated (95% air, 5% CO_2_, 37 °C, 24 h) to ensure they settled on the bottom of the plates. The MCF-7 or REF cells were exposed to galangin and GAL/β-CD, depending on IC_50_ value, for 24 h. After that, the medium was removed and collected. Test and control solutions were added at a volume of 100 µL per well. Except for the chromogen blanks, all samples were incubated with an anti-caspase-3 antibody solution (100 µL, 1 h, 25 °C). The same step was repeated for 30 min but this time with 1X Anti-Rabbit IgG HRP solution instead of anti-caspase-3 antibody. Samples were then treated with stabilized chromogen (100 µL), after which the color of the substrate solution started to turn blue. Incubation was then performed (30 min, room temperature, dark), followed by the addition of stop solution (100 µL). To ensure that their contents were well-mixed, plates were tapped, after which the color turned to yellow. Caspase-3 activity was measured by using an ELISA reader (450 nm) within 2 h after the addition of the stop solution [32].

### 2.14. In Vivo Assays

#### 2.14.1. Laboratory Mice

The naive male BALB/c mice (n = 21, age = 4–5 weeks, weight = 19–25 gm) were provided by the Iraqi Center for Cancer and Medical Genetic Researches, Mustansiriyah University, Baghdad, Iraq. One week prior to starting their treatment, mice were transferred to polyacrylic cages (7 mice/cage) with ad libitum food and water and standard maintenance conditions (RT, 55 ± 10 % relative humidity, 12:12 h light-dark cycle). All the protocols applied in these experiments were approved by the Animal Care and Ethics Committee, Biotechnology Division, Applied Sciences Department, University of Technology, Baghdad, Iraq, according to the Guidelines of the U.S. National Institutes of Health (NIH Publication No. 86–23, revised in 1996).

#### 2.14.2. Toxicity Assay 

A total of 21 Swiss albino male mice were divided into three groups (seven animals per group) as follows: group I—normal saline-treated control; group II—animals that received a low dose (20 mg Kg^−1^) of GAL/β-CD; group III—animals that received a high dose (640 mg Kg^−1^) of GAL/β-CD. The treated groups were orally administered for 14 days (single dose per day; 0.25 mL). Before giving the doses, as well as during the experimental period, the body weights of mice were recorded. Then, the blood, heart, liver, spleen, kidney, thymus, and lungs were collected immediately after sacrificing the animals. For serum collection, blood was centrifuged at 3000 rpm for 10 min, and the resulting serum was stored at −20 °C until used to estimate liver enzymes such as aspartate aminotransferase (AST/GOT), alkaline phosphatase (ALP), and alanine aminotransferase (ALT/GPT) and kidney function enzymes such as urea, creatinine, and uric acid, which were estimated in the serum by colorimetric analysis using a spectrophotometer manufactured by Spinreact, Spain. Spleen and thymus were stripped out and weighted, and their indices were calculated. The relative weight and the indices for the spleen and thymus were calculated according to the following equations.

(5) $$\mathrm{Relative}\;\mathrm{weight}\;\%\;=\frac{\mathrm{Weight}\;\mathrm{of}\;\mathrm{experimental}\;\mathrm{organ}\;}{\mathrm{Weight}\;\mathrm{of}\;\mathrm{the}\;\mathrm{experimental}\;\mathrm{animal}}$$

(6) $$\mathrm{Sx}=\frac{\mathrm{Weight}\;\mathrm{of}\;\mathrm{experiment}\;\mathrm{al}\;\mathrm{organ}\;/\;\mathrm{Weight}\;\mathrm{of}\;\mathrm{the}\;\mathrm{experiment}\;\mathrm{al}\;\mathrm{animal}\;}{\mathrm{Weight}\;\mathrm{of}\;\mathrm{the}\;\mathrm{control}\;\mathrm{organ}\;/\;\mathrm{Weight}\;\mathrm{of}\;\mathrm{the}\;\mathrm{control}\;\mathrm{animal}}$$

The targeted organs (thymus, liver, kidney, heart, spleen, and lung) were prepared for the histological study through several steps that included washing with PBS, fixation in 10% formalin and embedding in paraffin, sectioning by microtome, and staining with hematoxylin and eosin (H&E) [33].

### 2.15. Statistical Analysis

Significant differences (*p* ≤ 0.05) among the examined groups were tested via the application of the one-way analysis of variance (ANOVA, Tukey test). The results are expressed as mean ± standard deviation of three independent experiments. All statistical analyses were achieved by employing the Graph Pad Prism program (Version 6; Graph Pad Software Inc., La Jolla, CA, USA).

## 3. Results and Discussion

### 3.1. Preparation and Encapsulation Efficiency of GAL/β-CD

**Figure 1:**
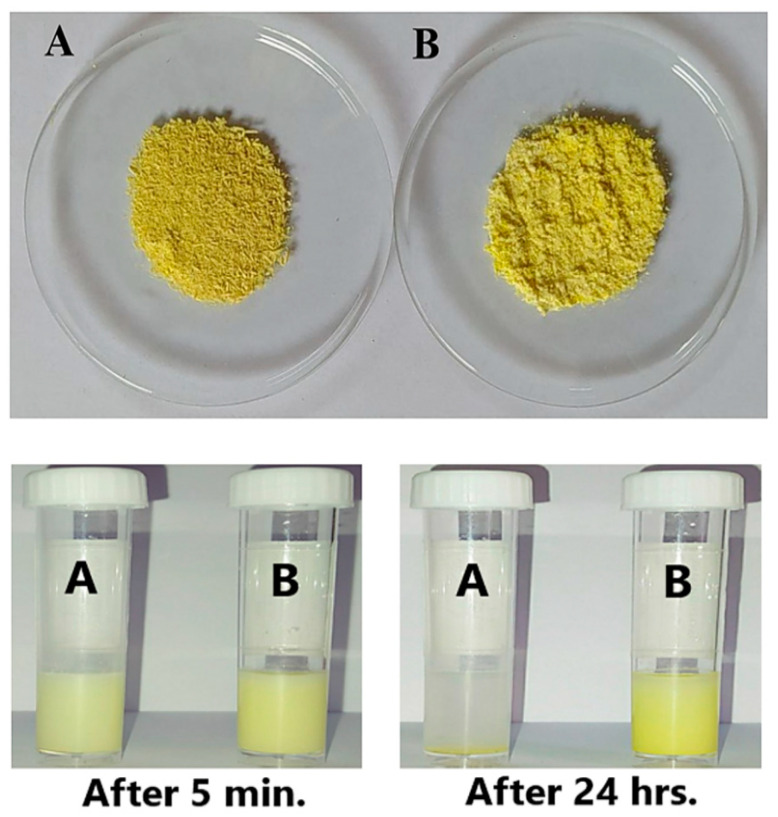
(Upper image) (**A**) Pure galangin and (**B**) GAL/β-CD powders and (lower image) the solubility of (**A**) pure galangin and (**B**) GAL/β-CD at 5 min and after 24 h of preparation.

Through the adoption of the nanoprecipitation method, GAL/β-CD inclusion complexes were made. Galangin is poorly water-soluble and susceptible to changes in pH and light, leading to limited oral absorption and difficulties in drug design and deployment [34]. As a result, we modified its delivery system and manufactured the GAL/β-CD using current pharmaceutical technologies. When the GAL/β-CD inclusion complex was dissolved in water, the powder formed a very fine dispersion and appeared to be soluble, unlike pure galangin, which is completely insoluble in water, with undissolved particles clearly visible in the suspension. Complete precipitation occurred in the original pure galangin after 24 h at room temperature. However, no such precipitation was observed in GAL/β-CD inclusion complexes (Figure 1). The results of the solubility investigation show that cyclodextrin enhances the water solubility of galangin. Because β-CD has a hydrophilic exterior and hydrophobic interior cavities, thermodynamic operating factors, including hydrogen bonding and van der Waals correlations, can induce it to form an inclusion complex with hydrophobic substances in a hydrophilic atmosphere [35,36]. The experimental results of Yao and his coworkers found that PEG modification effectively increases the solubility of galangin [37].

The recorded values of encapsulation efficiency and drug loading of the modified inclusion complex were 92.60% and 2.41%, respectively. These high values proved a strong affinity between the galangin and the β-CD. This might be because β-CD was able to efficiently load weakly water-soluble medicines into the cavity, increasing drug functional properties [38], which means that GAL/β-CD is more conducive to clinical applications.

### 3.2. In Vitro Release of GAL/β-CD

**Figure 2:**
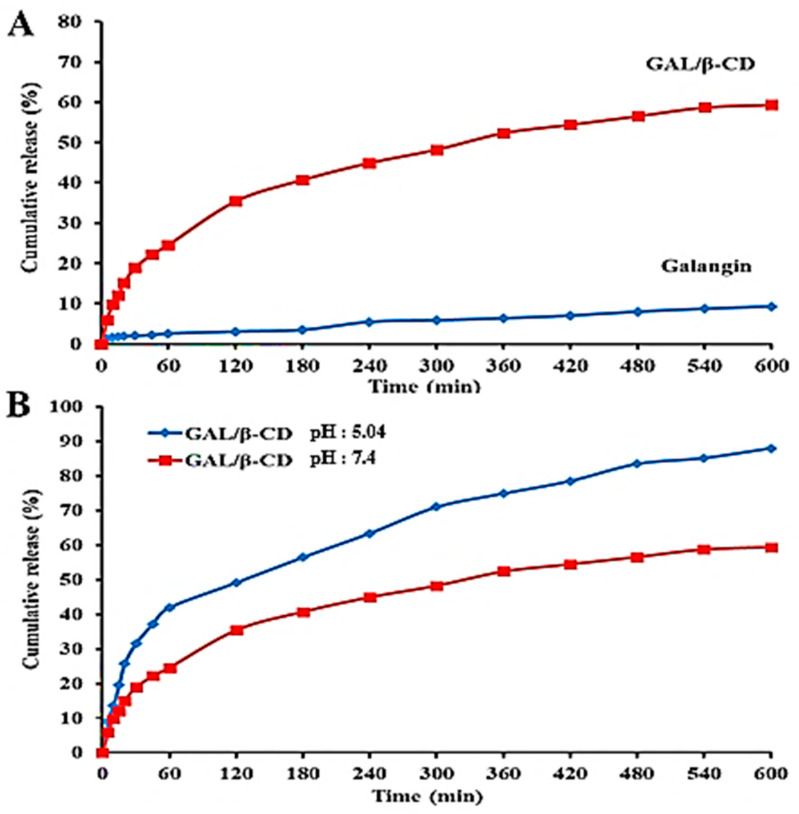
Release profile of (**A**) galangin and modified GAL/β-CD in PBS at pH 7.4 and (**B**) modified GAL/β-CD in PBS at pH 7.4 and 5.04.

The release of galangin and GAL/β-CD in physiological (pH = 7.4) and acidic (pH = 5.0) conditions was examined using UV–visible spectrophotometry, as shown in Figure 2. The in vitro release profile of galangin and GAL/β-CD is important for the delivery of drugs. Pure galangin showed a slow release of about only ~ 9.25% in physiological testing (pH = 7.4) (Figure 2A). The results demonstrated an inverse correlation between pH and the increase in the release rate of the modified GAL/β-CD, which was ~59.32% at pH 7.4 and ~87.97% at pH 5.04 within a period of 10 h (Figure 2B). GAL may be dissolved in an aqueous medium and meet the sink condition. Such reactions should undoubtedly affect galangin’s functional properties and control its release [39]. Whenever the medium dissolves into the circulation, the β-CD is hydrophilic, resulting in increased drug release. This was ascribed to β-CD’s capacity to transport hydrophobic medicines efficiently into its cavities [38].

### 3.3. UV–Vis Spectrum Analysis

**Figure 3:**
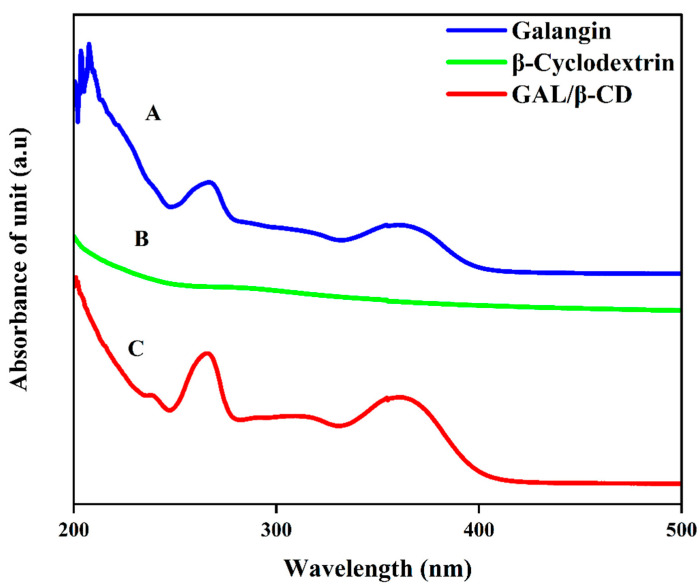
UV–vis spectroscopic analysis of (**A**) pure galangin, (**B**) β-cyclodextrin, and (**C**) GAL/β-CD.

The current UV–visible spectrophotometry data were used to confirm the conjugation of galangin and cyclodextrin and thus the formation of the GAL/β CD inclusion complex (Figure 3). Free galangin exhibits an intense maximum at 270 nm and a shoulder at 365, demonstrating the presence of different phenyl rings due to their π–π* electronic rearrangement and to the n–π* electronic transition of an electron from an n non-bonding molecular orbital to a π* anti-bonding molecular orbital, characteristic for the C=O bond (Figure 3A). The absorption spectrum of cyclodextrin is seen at 266 nm (Figure 3B). While the UV–vis spectra of galangin in the presence of β-CD, modifications in the absorbance values and bathochromic effects (when the wavelength maximum values are shifted from 270 to 272 nm and from 365 to 363 nm) were observed (Figure 3C), indicating that the GAL and β-CD were successfully combined into drug particles, leading to the modification of their individual photo-physical properties. The inclusion complex also contains methyl groups, which lead to the expansion of the hydrophobic area in the CD cavity and hence boost its affinity for galangin. Similar results were reported by Jullian, who found that βCD and its derivatives β-cyclodextrin (βCD), hydro xypropyl-β-cyclodextrin (HPβCD), or heptakis-2,6-O-di methyl-β-cyclodextrin (DMβCD) enhanced the water solubility of galangin [35].

### 3.4. Fourier Transforms Infrared Spectroscopy (FTIR) Analysis

**Figure 4:**
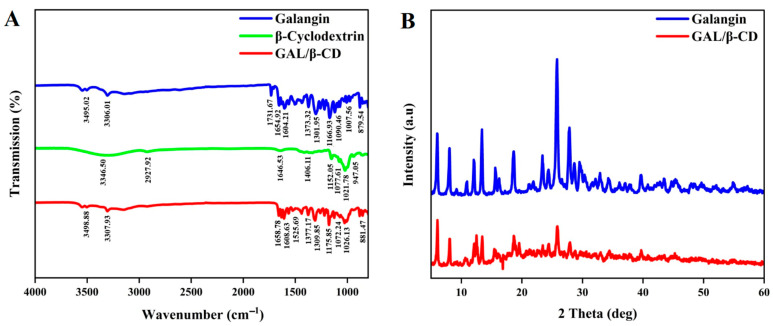
(**A**) Fourier transforms infrared spectroscopic analysis of pure galangin, β-cyclodextrin, and GAL/β-CD. (**B**) XRD of pure galangin and GAL/β-CD.

FTIR is among the methods used to measure infrared radiation’s spectral characteristics of wavelength and frequency, with quick and efficient identification of encapsulations of chemical molecules. The FTIR spectra of unbound galangin and the GAL/β-CD complex are presented in Figure 4A. Pure galangin’s FTIR spectra revealed distinct bands due to the presence of several functional groups, such as 3495.02, 3306.01, 1731.67, 1654.92, and 1166.93 cm^−1^, which could be ascribed to O-H stretching vibration, C=C, C=O, and C-O stretching, respectively. The FTIR spectrum of galangin has prominent peaks at 3426.89 and 1636.3 cm^−1^ that can be assigned to (O-H) and (C=O) stretchings, respectively [40]. The free flavonoids (GALs) are characterized by the sharp absorption band of the C=O stretching vibration, which appears at 1653 cm^−1^ [41]. The typical peaks of β-CD were found at 3400 cm^−1^ (–OH stretching), 2927 cm^−1^ (–CH stretching), 1400 cm^−1^ (–OH bending), and 950 cm^−1^ (skeleton movement that involves α-1, 4 linkage) [42]. Both the spectra of galangin and GAL/β-CD have the same distinctive peaks despite some differences. The characteristic bands of the carbonyl groups in β-cyclodextrin at 1071.83 cm^−1^ and 1026.28 cm^−1^ confirmed the successful conjugation and peak intensities in GAL/β-CD. Galangin peaks at 1090.46 cm^−1^ and 1007.56 cm^−1^ due to -C-O stretching were moved because of their implication in the hydrogen bonding occurring upon the trapping of the aromatic B-ring of galangin in the hydrophobic cavity of β-cyclodextrin. FTIR analysis was used to evaluate the chemical bonds and functional groups and characterize any chemical interactions that appeared in the polymer as a result of the addition of the drug during nanoparticle formulation [43].

### 3.5. X-ray Diffraction Characterization

**Figure 5:**
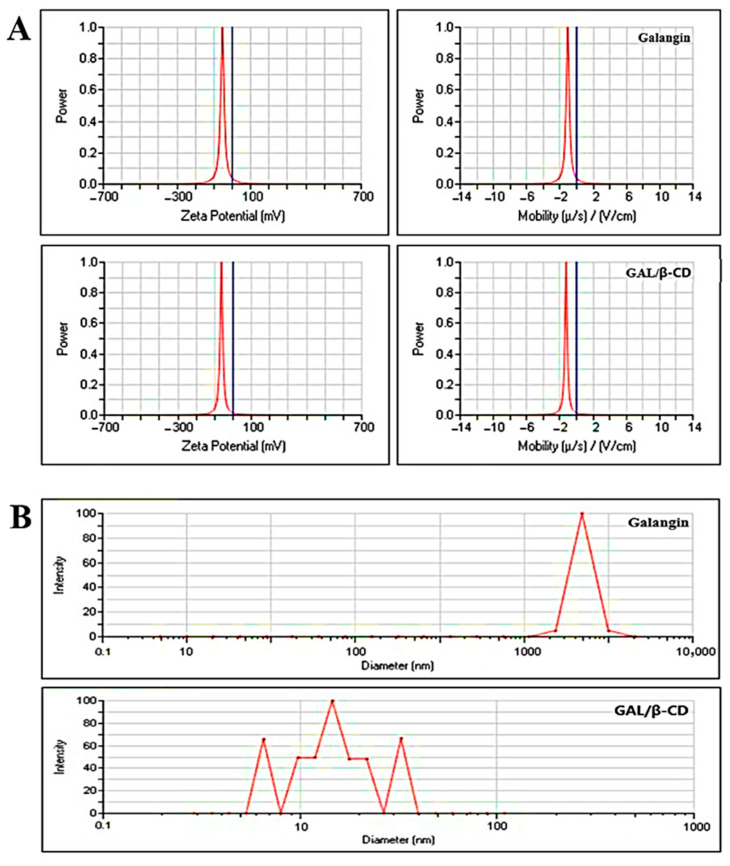
(**A**) Zeta potential analysis and (**B**) DLS analysis for pure galangin and GAL/β-CD.

The structures of galangin and GAL/β-CD were revealed by using XRD analysis (Figure 4B). XRD was applied for the evaluation of the crystalline status of galangin and GAL/β-CD. XRD is a useful tool for identifying the physical nature of particles. Free galangin has offered the powerful distinctive peaks of 2θ of 6.0°, 8.4°, 12.0 °, 13.3°, 15.6°, 18.6°, 23.4°, 25.7°, and 27.8°, suggesting a crystalline phase of high quality (Figure 5A). Patterns were also seen in GAL/β-CD, which had fewer peaks with varying absorption intensity values (Figure 5B). The hydrogen bonds between the hydroxyl groups of neighboring CD molecules were mediated by water molecules. There is credible evidence that the hydrogen bonds between CDs play an important role in stabilizing the complexes. X-ray diffraction is a rapid analytical technique primarily used for the measurement of atomic spacing. It provides accurate quantitative information on the atomic arrangements at interfaces, identifies the crystal phases present in the samples, and offers information on the size of unit cells with their physical properties [44,45].

### 3.6. Measurement of Zeta Potential and Particle Size

Results revealed that GAL/β-CD had a zeta potential value of −63.14 mV and a mobility value of −1.25 V/cm (Figure 5A). In contrast, the zeta potential value for pure galangin was found to be −53.49 mV, whereas the mobility value was −1.06 V/cm. The zeta potential is a critical physicochemical characteristic that affects nano-suspension stabilization. 

Very high or negative zeta potential values result in more repulsions, but the attraction between equivalent electrically charged molecules inhibits particle agglomeration and hence guarantees simple dispersion [46]. In general, potential values greater than 30 mV are associated with acceptable stability, whereas those greater than 60 mV are associated with exceptional stability [47]. Short-term stability is typically conferred by a potential value of 20 mV, whereas fast aggregation can be achieved in the range of −5 to 5 mV. The zeta potential value could also be used to identify whether a charged active substance is encapsulated within the NP’s core or on its outside [48]. The negative zeta potential value recorded in the present study indicates that the developed GAL/β-CD was electrically and physically stable. Cationic nanoparticles have been used by an increasing number of scientists because they provide a simple, safe, and effective vehicle to transport medicinal compounds [49].

Figure 5B shows the results of DLS analysis of galangin and GAL/β-CD. The computed average particle size of the formed GAL/β-CD was determined to be 23.9 nm, with a PDI value of approximately 0.387, based on dynamic light scattering measurements. In contrast, pure galangin had a wide size range, with an average particle size of around 9075.06 nm and a PDI value of 0.224. Interestingly, this difference can be the result of the drop in the surface free energy with increasing hydrophobicity, which results in a smaller particle size [40]. PDI is a number calculated from a two-parameter fit to the correlation data (the cumulating analysis). This index is dimensionless and scaled such that values smaller than 0.05 are mainly seen with highly monodisperse standards. PDI values bigger than 0.7 indicate that the sample has a very broad particle size distribution and is probably not suitable to be analyzed by DLS [50].

### 3.7. Field Emission Scanning Electron Microscopy 

**Figure 6:**
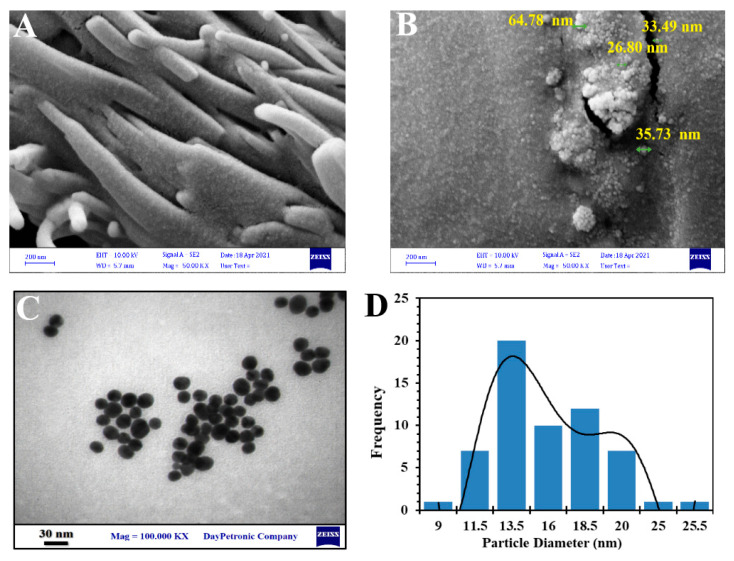
Image analysis of electron microscope (FESEM) of (**A**) pure galangin and (**B**) GAL/β-CD, (**C**) transmission electron microscopy (TEM), and (**D**) particle size distribution.

The FE-SEM was performed to study the size and morphology of galangin and GAL/β-CD. As shown in Figure 6A,B, the original galangin powder exhibited particles that lacked uniformity in size, appeared to be aggregated, and had an irregular arrangement. GAL/β-CD revealed granules that were nicely spaced and clear, with less crystallinity and an absence of larger particles. Their diameter was in the range of 32 nm, while almost every particle was dispersed as a single molecule, with clumped distributions also being visible. These results validate those of Ferraz et al., who reported the spherical form of these particles in FESEM images [51].

### 3.8. Transmission Electron Microscopy 

The TEM assay has been widely used to obtain information regarding the shape and size of polymeric NPs [52]. As depicted in Figure 6C,D, TEM analysis shows that GAL/β-CD complexes have a spherical shape, crystalline nature, flat surface, and a more exact match layout, with a diameter of 13.5 nm. The results of TEM reveal that galangin is homogeneously distributed on the surface of β-cyclodextrin to form an inclusion complex. 

### 3.9. Serum Stability Study

**Figure 7:**
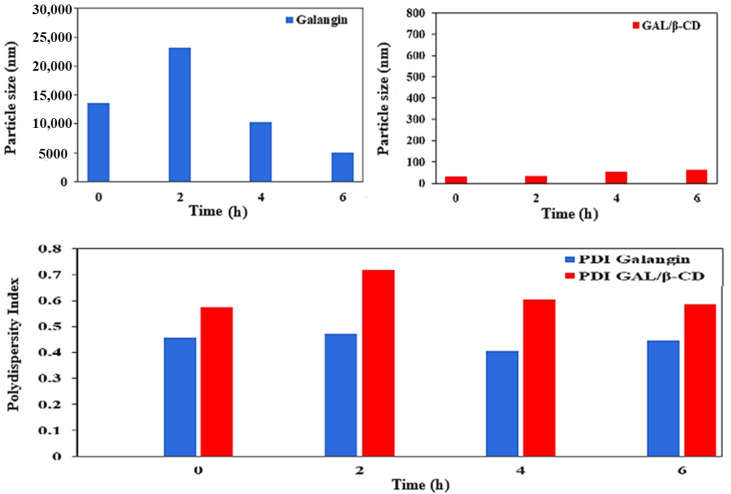
Serum stability of pure galangin and GAL/β-CD for 6 h.

Serum stability is an important assay for any oral medication-delivery device for preclinical research protein interactions of the inclusion complex. Thus, dynamic light scattering was utilized to document the time-related alterations in the values of PS and PDI of galangin and GAL/β-CD after incubation with 10% FBS. Figure 7 demonstrates a drastically decreased PS value for galangin following its initial combination with 10% FBS (13,591 vs. 5003 nm); it was visible 2 h after incubation. Subsequently, the PS of galangin dropped dramatically, even to levels below those seen prior to incubation with 10% FBS. It was discovered that serum proteins interact with the distribution of nanoparticles in two ways. The first mechanism involves the creation of a protein corona as a result of serum proteins adhering to nanoparticle surfaces. In fact, the initial rise in galangin size observed in the present study was most likely caused by this protein corona. In the second mechanism, serum proteins may impact nanoparticulate dispersions by their induction of osmotic pressure. Water escapes from the aqueous core due to the osmotic effect of these proteins, causing the nanoparticles to shrivel [28]. The osmotic pressure of a solution is caused by the decrease in the solvent’s chemical potential due to the presence of solutes [53]. The serum-induced shrinking of galangin after 2 h incubation in FBS solution might have been caused by the latter effect. In contrast, no significant changes in PS or PDI values of GAL/β-CD were seen throughout time, suggesting that they were stable in serum for 6 h. The PS of GAL/β-CD ranged from 30 to 63.9 nm. This finding supports the hypothesis that β-cyclodextrin formation of polyelectrolyte layers causes forces of repulsion against interactions with serum protein, shielding GAL/β-CD from being opsonized and subsequently cleared by the rough endoplasmic reticulum system. For future delivery of drugs, a previous study reported the use of a poly electrolyte multilayer film based on cationic and anionic β-cyclodextrin polyelectrolytes that was placed onto a textile material [54].

### 3.10. Blood Compatibility Assay

**Figure 8:**
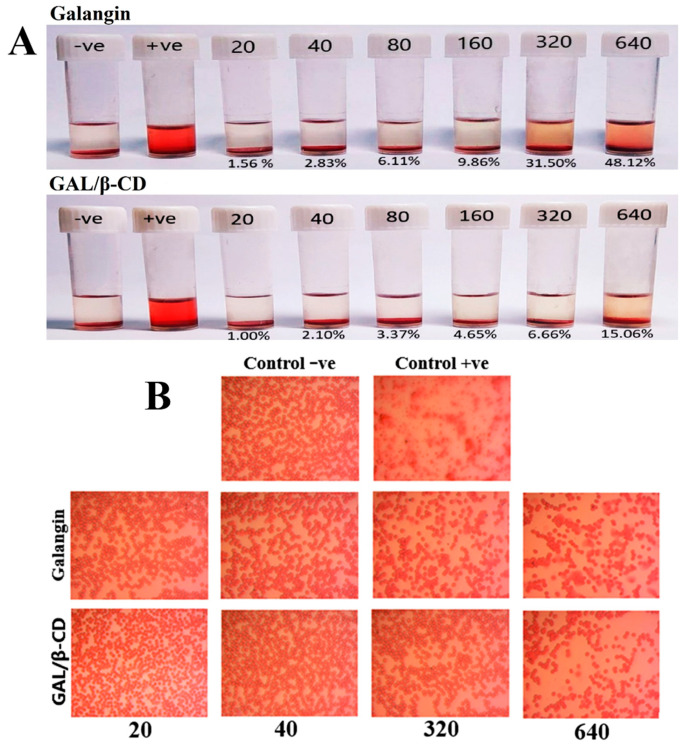
(**A**) Photograph of human RBCs indicating the hemolysis ratio following treatment with pure galangin and modified GAL/β-CD at different concentrations (20, 40, 80, 160, 320, and 640 µg mL^−1^) and (**B**) light microscopic images showing the hemolytic toxicity of pure galangin and GAL/β-CD at concentration 20, 40, 320, and 640 µg mL^−1^, respectively. (magnification power: 40×). Control −ve, RBCs incubated with normal saline; Control +ve, RBCs incubated with D.W.

Figure 8A shows the effects on RBC compatibility and hemolytic activities following treatment with six concentrations of pure galangin or GAL/β-CD (20, 40, 80, 160, 320, and 640 µg mL^−1^). Distilled water and normal saline were applied as positive and negative controls, respectively. The results indicated that GAL/β-CD has fewer effects on RBCs than pure galangin. The hemolysis percentage values resulting from the treatment with GAL/β-CD at the concentrations of 20, 40, 80, and 160 µg mL^−1^ did not exceed the permissible threshold of less than 5% hemolysis, which were 1.00%, 2.10%, 3.37%, and 4.65%, respectively, while the hemolysis values in response to treatment with 320 and 640 µg mL^−1^ exceeded this level by 6.66% and 15.06%, respectively. The observed blood compatibility of GAL/β-CD may be attributed to increasing the electrostatic repulsion on the surface due to the presence of similar charges (negative charges) and to the inclusion of galangin inside the cyclodextrin cavity.

In contrast, the higher concentrations of unbound galangin (80, 160, 320, and 640 µg mL^−1^) caused hemolysis that was higher than the permissible level (6.11%, 9.86%, 31.50%, and 48.12%, respectively); however, hemolysis caused by the lower concentrations of 20 and 40 µg mL^−1^ was within the permissible level (1.56% and 2.83%, respectively). Due to the obviously high content of membranous unsaturated fatty acids and cellular oxygen and hemoglobin, erythrocytes exert a particularly high vulnerability to oxidative damage [55]. Earlier findings showed that quercetin can protect human and animal RBCs from hemolytic destruction in vitro [56]. This influence was associated with a number of mechanisms, such as those related to maintaining membrane integrity, altering membranous proteins and lipids, and changing cell membrane water content [57]. The hemolytic activity of β-cyclodextrin (β-CD) on rabbit erythrocytes was reduced by the introduction of negatively charged groups onto the hydroxyls of β-CD; the membrane disrupting abilities decreased. In a previous study by Mazzarino on RBC, nanoparticles had less effect on erythrocyte hemolysis, indicating good blood compatibility [58]. 

The standard no. E2524-08 entitled “Test Method for Analysis of Hemolytic Properties of Nanoparticles” issued by the American Society for Testing and Materials (ASTM) states that hemolysis should not be higher than 5%; otherwise, the tested material is considered deteriorative to RBCs [59]. Based on this standard, the GAL/β-CD tested in the present study are hemo-compatible. The results of light microscopic imaging of RBCs shown in Figure 8B elucidate the effects of galangin and GAL/β-CD at lower and higher concentrations. The hemolysis experiment indicated no unfavorable effects on RBC shape over the examined lower range of concentrations of GAL/β-CD. Unbound galangin, on the other hand, had more harmful effects on RBCs, causing damage to cellular membranes, with this effect being similar to that of the positive control.

### 3.11. Cytotoxicity and Viability Tests

**Figure 9:**
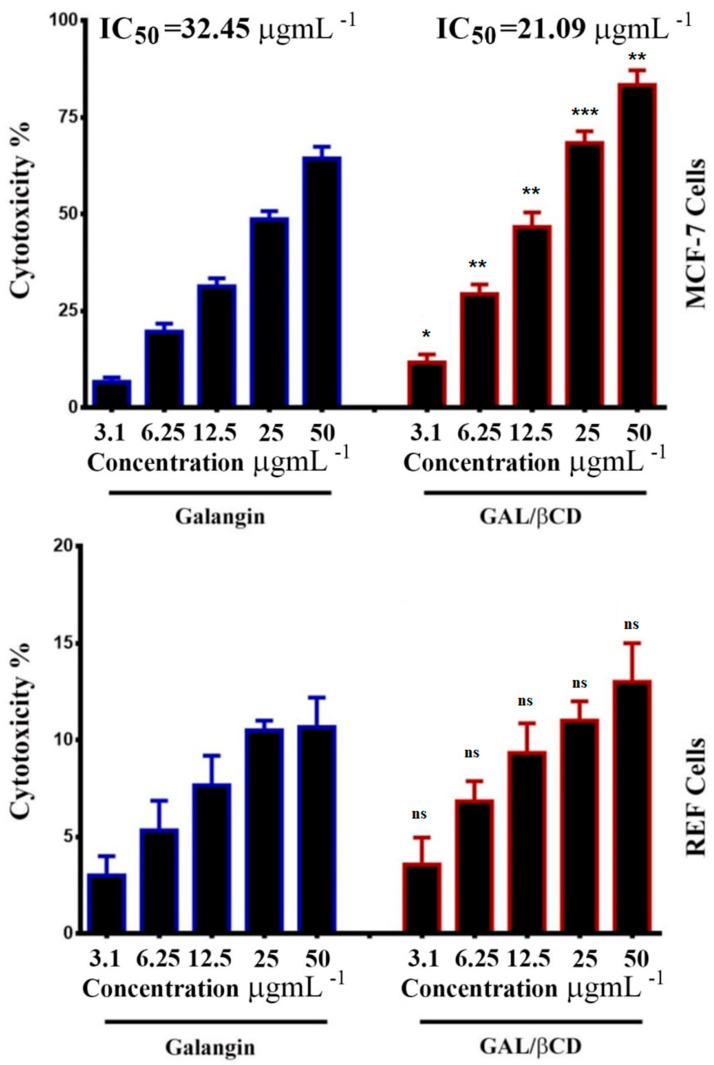
Growth inhibition of MCF-7 human BC and REF normal cell lines treated with different concentrations (3.1, 6.25, 12.5, 25 and 50 μg mL^−1^) of galangin and GAL/β-CD. * *p* ≤ 0.05, ** *p* ≤ 0.01, *** *p* ≤ 0.001. ns, non-significant.

One of the most widely used compounds in colorimetric assays for determining cytotoxicity or cell viability by testing the effects of mitochondrial enzymes, such as succinate dehydrogenase, is MTT (3-(4,5-dimethylthiazol-2-yl)-2–5-diphenyltetrazolium bromide) [60]. The cell viability of the tested cell lines in response to treatment with galangin and GAL/β-CD was detected by this test (Figure 9). MTT is converted to insoluble purple MTT-formazan crystals when it enters metabolically active cells, which are subsequently dissolved in a solvent and spectrophotometrically evaluated [61]. In the present study, the GAL/β-CD inclusion complex exhibited higher activity against MCF-7 in comparison to that exerted by galangin. As seen in Figure 9, there was dose-dependent cellular toxicity when cells were treated with the GAL/β-CD. The highest cytotoxicity (83.33%) was observed after treatment with 50 µg mL^−1^ of GAL/β-CD, whereas the cytotoxicity was 11.67% at 3.1 µg mL^−1^. Galangin caused smaller cytotoxicity values of 64.33% and 6.66%, respectively. This impact of galangin on BC cells might reflect a proliferation inhibitory mechanism by inducing apoptosis [62]. The β-CD polymer could be developed as a safe nanocarrier in drug-delivery systems and thus increase the anticancer activity of drugs against MCF7 cells [63]. In contrast, no such activity was observed in rat embryo fibroblast (REF). The proliferation rate of normal cells demonstrated less cytotoxic effects when compared with those observed in cancer cells.

### 3.12. Crystal Violet Staining Method

**Figure 10:**
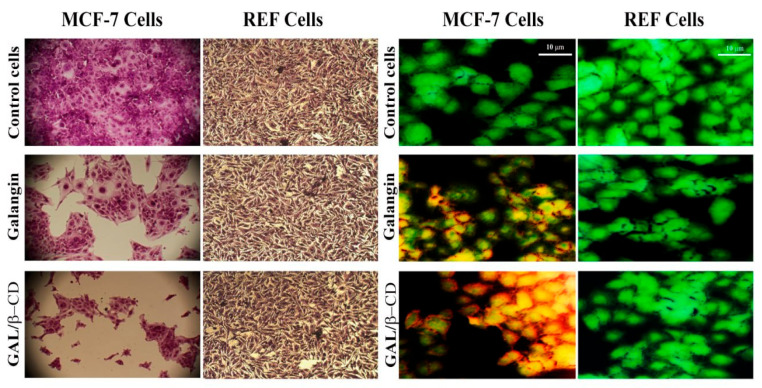
Microscopic images of MCF-7 cell line and REF cell line after treatment with IC_50_ of galangin and GAL/β-CD. (**Left** lane) staining with crystal violet dye (magnification power: 40×). (**Right** lane) acridine orange–ethidium bromide dual staining assay (Scale bar 10 µm).

The cytotoxicity effect of pure galangin and GAL/β-CD on the tested cells was also examined by applying the (Figure 10, Left lane). The results indicated that galangin alone has lower cytotoxicity to MCF-7 cells. However, GAL/β-CD exerted more severe toxic impacts on MCF-7 cells in a concentration-dependent manner. No such alterations were seen in normal cells. The proliferation rate of normal cells demonstrated fewer cytotoxic effects when compared with those observed in cancer cells. The crystal violet test is based on the affinity between the dye and the exterior surface of the DNA double helix. The quantity of dye absorbed is proportional to the total DNA content of the culture, allowing the number of viable cells to be estimated [64]. The results also demonstrated that GAL/β-CD resulted in several other changes, including cell morphology modifications, clustering of treated cells with few cellular extensions, and cell communication blocking. Cells that undergo cell death lose their adherence and are subsequently lost from the population of cells, reducing the amount of crystal violet staining in a culture. However, less alteration was seen in cells treated with pure galangin, while no similar impacts were recorded in untreated cells (Figure 10, left lane). Due to their smaller size, nanoparticles can infiltrate cells more efficiently than microparticles, making it easier to administer greater doses of medicine to achieve better results. Importantly, it is potentially possible to overcome the first-pass metabolism and P-glycoprotein-mediated drug efflux by encapsulating anticancer drugs in polymeric nanoparticles [65]. Various studies suggest that the cytotoxic effects of nanoparticles toward cancer cells could be related to the membrane-disrupting, accumulate of lactic acid, ROS, DNA damage, and apoptosis-inducing activities [66,67].

### 3.13. Double Staining with Acridine Orange–Ethidium Bromide 

The potentials of the IC_50_ preparations of pure galangin and the modified GAL/β-CD to induce cytotoxicity were examined through the application of the dual staining test with AO and EtBr dyes in both MCF-7 and REF cells (Figure 10, right lane). The changes in nuclear morphology were analyzed and further confirmed by using AO/EtBr dual staining fluorescence microscopy in MCF-7 cells. When these cells were treated with GAL/β-CD, they showed a more dramatic breakdown of membrane stability as compared to untreated cells. Fewer effects were observed in cells treated with galangin. The examination of alterations in fluorescence and morphology of treated cells provided a valuable tool for the efficient recognition of the remarkably induced alterations, including chromatin condensation and the appearance of red staining in the cytoplasm, which reflect alterations in RNA and lysosomes in cells exposed to drugs. The nuclei of early apoptotic cells exhibit a yellow color, while their chromatin is characterized either by condensation or fragmentation. Nevertheless, the nuclei of late apoptotic cells are orange to red in color, whereas their chromatin is either condensed or broken. AO/EtBr based analysis of the nuclear morphology of cells tested in the present study revealed apoptotic cell death. It is worth noting that both live and dead cells can be stained by AO. However, EtBr is strictly utilized to stain the DNA of cells with damaged membrane integrity. It was previously shown that the living cells (green color) had a homogeneous distribution and huge nuclei, while the treated cells were recorded as dead (red color), indicating that it had a cytotoxic impact [68].

### 3.14. Mitochondrial Activity

**Figure 11:**
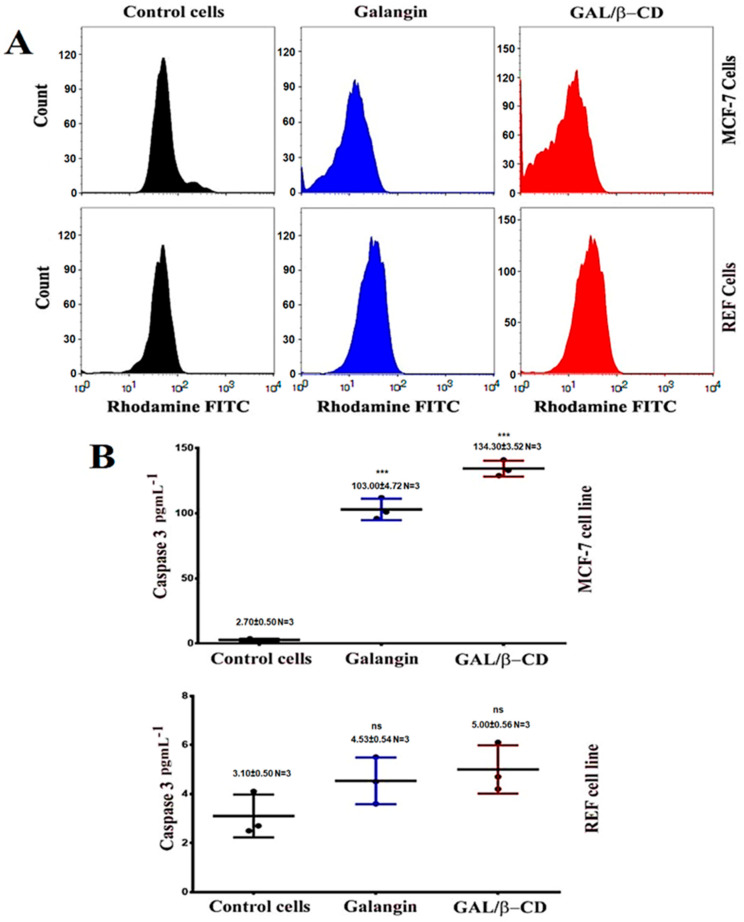
(**A**) Flow cytometry data of MCF-7 and REF cells treated as indicated. Rhodamine stain was used to investigate loss of mitochondrial activity. (**B**) Caspase-3 protein induction in MCF-7 and REF cells. *** *p* ≤ 0.001. ns, non-significant. N = 3, three replicates.

For further investigation, a flow cytometry method was performed to detect apoptosis (Figure 11A). The reduced membrane potential of the mitochondria is regarded as a clear indicator of cell death by apoptosis. After labeling MCF-7 and REF cells with the Rh123 probe, flow cytometry was utilized to measure the changes in this potential. As shown in Figure 11A, the number of apoptotic MCF-7 cells demonstrated a significant rise after their exposure to the tested substances. The 24 h treatment of MCF-7 cells with galangin and modified GAL/β-CD resulted in a fundamental reduction in the level of staining with Rh123, indicating a drop in the membrane potential of the mitochondria in comparison with the untreated MCF-7 cells. The data imply that alterations in the mitochondrial mediated apoptosis pathway may cause apoptosis in modified GAL/β-CD cells. Galangin caused fast depolarization of the mitochondrial membrane, which led to hypopolarization. Furthermore, the current work used a mitochondrion-selective fluorescent packaged protein (pDsRed2-Mito), whose absorption is dependent on intact mitochondrial fragmentation, to see if galangin damages the mitochondria. Mitochondrial fragmentation is a well-known phenomenon that occurs after the start of apoptosis and is involved in the apoptosis-related activation of outer mitochondrial membrane scission during cell death [69].

### 3.15. ELISA Assays

The goal of this experiment was to evaluate if GAL/β-CD causes apoptosis in MCF-7 cells by increasing the detection of caspase-3 proteins. The findings are shown in Figure 11B. Following treatment with galangin, there was a small rise in caspase-3 detection, but after treatment with GAL/β-CD, the detection was greater. As a result, the accumulation of caspase-3 proteins in MCF-7 cells suggested that GAL/β-CD might increase MCF-7 cells’ sensitivity to apoptosis via promoting caspase-3 singling mechanisms. Consequently, both extrinsic and intrinsic apoptotic routes resulted in caspase-3 activation during apoptosis. Caspase-3 plays an important part in apoptosis. It is also considered for nucleic acids to be fragmented, which results in the apoptotic DNA ladder pattern [70]. Galangin affects the cell cycle during its G0/G1 phase, modulates cyclin/cdk expression, and causes apoptosis. Galangin was also shown to boost the cleaved caspase-3 expression [71]. The cleavage of caspase-3 and PARP was shown to be enhanced as a result of galangin therapy, contributing to the apoptotic death of TU212 and HEP-2 cells [72]. However, less activity was seen in REF when compared with that observed in MCF-7 cells.

### 3.16. In Vivo Toxicity Assay

**Figure 12:**
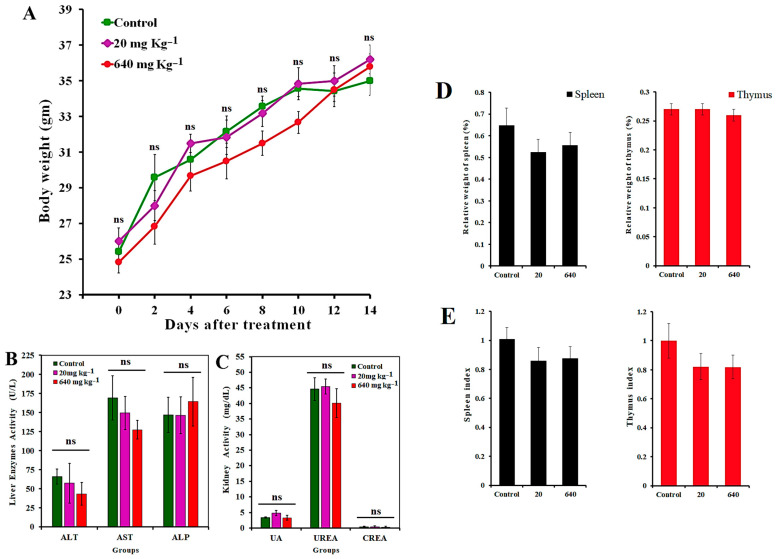
(**A**) Effect of GAL/β-CD on mice body weight. No significant difference was detected in both dosage 20 mg kg^−1^ and 640 mg kg^−1^. Each value represents mean ± SEM. (**B**,**C**) Serum levels of liver and kidney function parameters in mice treated with GAL/β-CD. Liver enzymes: ALT, alanine transaminase; ALP, alkaline phosphatase; AST, aspartate transaminase. Kidney function parameters: uric acid. Urea kidney function parameters: creatinine. Data are expressed as mean ± SEM. ns, non-significant. (**D**,**E**) The relative weight and indices of spleen and thymus after oral administration with GAL/β-CD for 14 days by different doses (20 mg kg^−1^, 640 mg kg^−1^).

Figure 12A depicts how mice’s body weight changed after being given two different doses of GAL/β-CD. It can be shown that the 2-week treatment of mice with GAL/β-CD in the dose range of 20 to 640 mg kg^−1^ did not lead to their death. Moreover, body weight values of GAL/β-CD-treated and control mice did not show significant differences (*p* ≤ 0.05). Moreover, both groups did not exhibit signs of aberrant alterations, both clinically and behaviorally, and GAL/β-CD therapy did not appear to cause any harm in the treated animals, which maintained good health status to the end of the study. There were also no clinical signs or symptoms, such as redness, swelling, difficulty moving, hunching, or strange behaviors [73,74].

It is also necessary to investigate whether GAL/β-CD treatment leads to toxic effects on organs such as the liver and kidney. The levels of biochemical parameters in the serum of treated mice were analyzed, including ALT, AST, ALP, blood urea nitrogen, creatinine, and uric acid (Figure 12B,C). Urea, creatinine, and uric acid are products of metabolism that are used to indicate the level of functioning of the kidney. In addition, liver function can be indicated via testing serum levels of ALT, AST, and ALP. The differences in the levels of these parameters between the tested groups were found to be non-significant (*p* ≤ 0.05) when compared with the control group. The changes in the levels of ALT, AST, and CERA are still not clear if the mice are treated for a long time, a result that is identical to that presented by Jasper et al. [75].

To further investigate the effects of GAL/β-CD at two doses on the immune reactions in the lymph organs, the relative weight and indices of organs for the spleen and thymus of mice were collected (Figure 12D,E). The values of spleen index in mice treated with 20 and 640 mg kg^−1^ were 0.86 ± 0.09 and 0.87 ± 0.08, respectively, whereas those for the thymus were 0.82 ± 0.06 and 0.82 ± 0.09, respectively. It can be seen that the values of the indices of the spleen and thymus had no significant differences among the studied groups. Spleen and thymus indices are important indicators of immune system function in mice [76,77].

**Figure 13:**
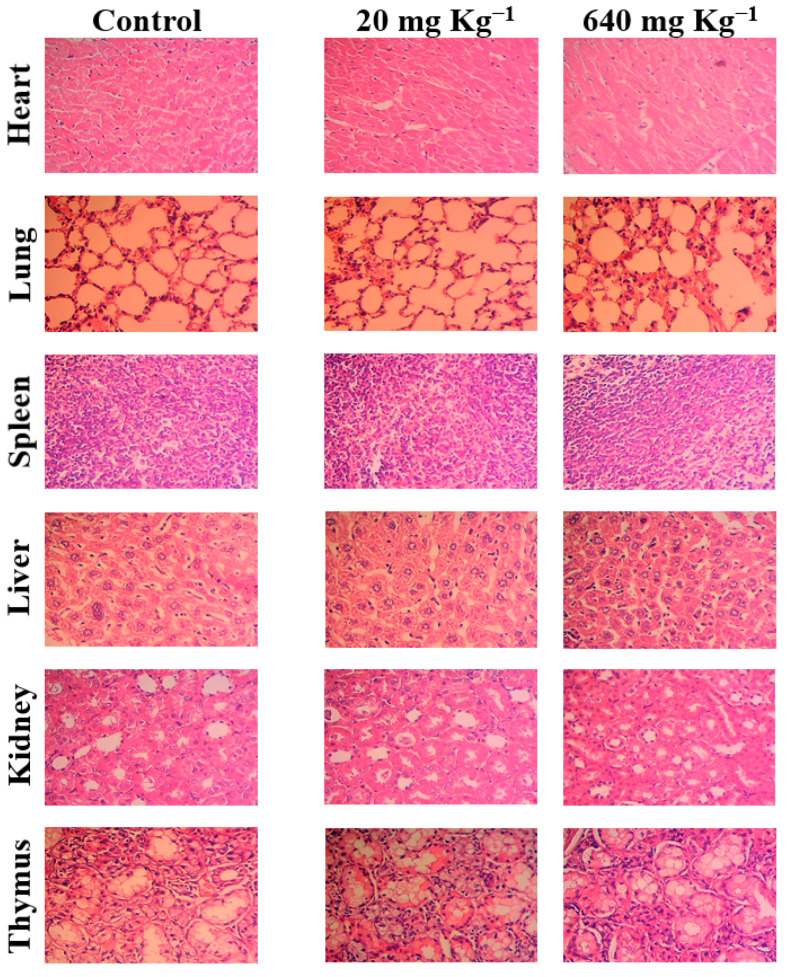
Histological images for heart, lung, liver, spleen, kidney, and thymus of mice orally treated with different doses of GAL/β-CD (20 mg kg^−1^, 640 mg kg^−1^), (magnification power: 40×).

The tissues of the liver, kidneys, heart, lung, thymus, and spleen of mice of both treated and untreated groups were subjected to histopathological examination aimed at evaluating the possibility of any tissue damage, inflammation, or lesions in response to treatment with GAL/β-CD. Figure 13 illustrates that the mice treated with GAL/β-CD showed neither visible signs of damage nor histological abnormalities. The hepatic cords and hepatic lobules of the animals from various treatment groups exhibited no significant differences. Similarly, the lymphocyte counts in the splenic lymphoid nodules did not show a significant reduction.

The treated groups’ kidney tissues were similar to those of the control group because the glomerular and renal tubular epithelial cells did not suffer from atrophy. While lung tissue slices revealed no significant congestion of pulmonary capillaries with erythrocytes, isolated intra-alveolar bleeding, or mononuclear cell infiltration, no other alterations in lung micromorphology were noted (e.g., alveolar septal thickening or hypercellularity). The cardiac muscle, i.e., the myocardium, is made up of cross-striated muscle cells called cardiomyocytes, with one central nucleus. Intercalated discs, which are specialized junctions between cardiac cells, were seen in cardiac muscles with no enhanced cytoplasmic vacuolization or myofibrillar loss. As a result, animals exposed to GAL/β-CD showed no histopathological abnormalities in their liver, spleen, lung, kidney, heart, and thymus, indicating that employing GAL/β-CD for in vivo applications is safe. At the conclusion of the experiment, there were no macroscopic organ alterations in the treated groups. A previous study on the flavonoid hesperidin reported that the treated mice did not suffer from damaging impacts or histopathological alterations [29]. Furthermore, it has been shown that galangin has no toxic effect on the histology of mice and reduces the toxic effect of paracetamol injection [78].

## 4. Conclusions

Several methods for the characterization of GAL/β-CD inclusion complexes have been developed. When tested on human red blood cells and fetal bovine serum, GAL/β CD was found to be cytocompatible, therapeutically efficient, safe, and more effective in comparison with pure galangin. GAL/β-CD also did not lead to any apparent adverse effects, organ injury, or behavioral distortions in the treated animals. These results confirm that GAL/β-CD possesses powerful cytotoxicity and can increase human BC cell death via the apoptotic mechanism of caspase-without affecting normal tissues. Thus, synthesized GAL/β-CD is vital for a system that can sufficiently reach all targeted sites without creating major negative consequences. These results showed promising potential for new liquid formulations of galangin using β-CD, which can be used to improve the properties of galangin and might provide inspiration for further application of an inclusion complex in food, pharmaceutical, and medicine fields in the future.

## Data Availability

Data are available in the manuscript.

## Sample Availability

Samples of the compounds are available from the authors.

## References

[1] Bazak R., Houri M., El Achy S., Kamel S., Refaat T. (2015). Cancer active targeting by nanoparticles: A comprehensive review of literature. J. Cancer Res. Clin. Oncol..

[2] Siegel R.L., Miller K.D., Fedewa S.A., Ahnen D.J., Meester R.G., Barzi A., Jemal A. (2017). Colorectal cancer statistics, 2017. CA Cancer J. Clin..

[3] Sung H., Ferlay J., Siegel R.L., Laversanne M., Soerjomataram I., Jemal A., Bray F. (2021). Global cancer statistics 2020: GLOBOCAN estimates of incidence and mortality worldwide for 36 cancers in 185 countries. CA Cancer J. Clin..

[4] Plummer M., de Martel C., Vignat J., Ferlay J., Bray F., Franceschi S. (2016). Global burden of cancers attributable to infections in 2012: A synthetic analysis. Lancet Glob. Health.

[5] Miller K.D., Fidler-Benaoudia M., Keegan T.H., Hipp H.S., Jemal A., Siegel R.L. (2020). Cancer statistics for adolescents and young adults, 2020. CA Cancer J. Clin..

[6] Engelhardt E.G., Kriege M., Hooning M.J., Seynaeve C., Tollenaar R.A., van Asperen C.J., Ausems M.G.E.M., van de Poll-Franse L.V., Mook S., Verhoef S. (2015). Familial versus sporadic breast cancer: Different treatments for similar tumors?. Adv. Breast Cancer Res..

[7] Xu W., Neckers L. (2017). A USE1ful biomarker and molecular target in Lung Cancer?. JNCI J. Natl. Cancer Inst..

[8] Burguin A., Diorio C., Durocher F. (2021). Breast Cancer Treatments: Updates and New Challenges. J. Pers. Med..

[9] Yonekura-Sakakibara K., Higashi Y., Nakabayashi R. (2019). The origin and evolution of plant flavonoid metabolism. Front. Plant Sci..

[10] Wen W., Alseekh S., Fernie A.R. (2020). Conservation and diversification of flavonoid metabolism in the plant kingdom. Curr. Opin. Plant Biol..

[11] Rauter A.P., Ennis M., Hellwich K.H., Herold B.J., Horton D., Moss G.P., Schomburg I. (2018). Nomenclature of flavonoids (IUPAC recommendations 2017). Pure Appl. Chem..

[12] Imran M., Rauf A., Abu-Izneid T., Nadeem M., Shariati M.A., Khan I.A., Imran A., Orhan I.E., Rizwan M., Atif M. (2019). Luteolin, a flavonoid, as an anticancer agent: A review. Biomed. Pharmacother..

[13] Patil S., Ujalambkar V., Rathore A., Rojatkar S., Pokharkar V. (2019). Galangin loaded galactosylated pluronic F68 polymeric micelles for liver targeting. Biomed. Pharmacother..

[14] Lee J.J., Lee J.H., Yim N.H., Han J.H., Ma J.Y. (2017). Application of galangin, an active component of Alpinia officinarum Hance (Zingiberaceae), for use in drug-eluting stents. Sci. Rep..

[15] Zhu J., Wang Q., Li H., Zhang H., Zhu Y., Omari-Siaw E., Sun C., Wei Q., Deng W., Yu J. (2018). Galangin-loaded, liver targeting liposomes: Optimization and hepatoprotective efficacy. J. Drug Deliv. Sci. Technol..

[16] Li X., Wang Y., Xiong Y., Wu J., Ding H., Chen X., Lan L., Zhang H. (2016). Galangin Induces Autophagy via Deacetylation of LC3 by SIRT1 in HepG2 Cells. Sci. Rep..

[17] Wang Y., Lin B., Li H., Lan L., Yu H., Wu S., Wu J., Zhang H. (2017). Galangin suppresses hepatocellular carcinoma cell proliferation by reversing the Warburg effect. Biomed. Pharmacother..

[18] Tian B., Hua S., Liu J. (2020). Cyclodextrin-based delivery systems for chemotherapeutic anticancer drugs: A review. Carbohydr. Polym..

[19] Liu Q., Zhou Y., Lu J., Zhou Y. (2020). Novel cyclodextrin-based adsorbents for removing pollutants from wastewater: A critical review. Chemosphere.

[20] Sarkar B., Mahanty A., Gupta S.K., Choudhury A.R., Daware A., Bhattacharjee S. (2022). Nanotechnology: A next-generation tool for sustainable aquaculture. Aquaculture.

[21] Jin C., Wang K., Oppong-Gyebi A., Hu J. (2020). Application of nanotechnology in cancer diagnosis and therapy-a mini-review. Int. J. Med. Sci..

[22] Tripathi S., Sanjeevi R., Anuradha J., Chauhan D.S., Rathoure A.K. (2022). Nano-bioremediation: Nanotechnology and bioremediation. Research Anthology on Emerging Techniques in Environmental Remediation.

[23] Ali Z., Jabir M., Al-Shammari A. (2019). Gold nanoparticles inhibiting proliferation of Human breast cancer cell line. Res. J. Biotechnol..

[24] Kiio T.M., Park S. (2021). Physical properties of nanoparticles do matter. J. Pharm. Investig..

[25] Al-Jubori A.A., Sulaiman G.M., Tawfeeq A.T., Mohammed H.A., Khan R.A., Mohammed S.A. (2021). Layer-by-Layer Nanoparticles of Tamoxifen and Resveratrol for Dual Drug Delivery System and Potential Triple-Negative Breast Cancer Treatment. Pharmaceutics.

[26] Zhang L., Man S., Qiu H., Liu Z., Zhang M., Ma L., Gao W. (2016). Curcumin-cyclodextrin complexes enhanced the anti-cancer effects of curcumin. Environ. Toxicol. Pharmacol..

[27] Mora L., Chumbimuni-Torres K.Y., Clawson C., Hernandez L., Zhang L., Wang J. (2009). Real-time electrochemical monitoring of drug release from therapeutic nanoparticles. J. Control. Release.

[28] Freag M.S., Elnaggar Y.S., Abdelmonsif D.A., Abdallah O.Y. (2016). Layer-by-layer-coated lyotropic liquid crystalline nanoparticles for active tumor targeting of rapamycin. Nanomedicine.

[29] Sulaiman G.M., Waheeb H.M., Jabir M.S., Khazaal S.H., Dewir Y.H., Naidoo Y. (2020). Hesperidin Loaded on Gold Nanoparticles as a Drug Delivery System for a Successful Biocompatible, Anti-Cancer, Anti-Inflammatory and Phagocytosis Inducer Model. Sci. Rep..

[30] Joshy K.S., Sharma C.P., Kalarikkal N., Sandeep K., Thomas S., Pothen L.A. (2016). Evaluation of in-vitro cytotoxicity and cellular uptake efficiency of zidovudine-loaded solid lipid nanoparticles modified with Aloe Vera in glioma cells. Mater. Sci. Eng. C.

[31] Moyano D.F., Goldsmith M., Solfiell D.J., Landesman-Milo D., Miranda O.R., Peer D., Rotello V.M. (2012). Nanoparticle hydrophobicity dictates immune response. J. Am. Chem. Soc..

[32] Jabir M., Sahib U.I., Taqi Z., Taha A., Sulaiman G., Albukhaty S., Al-Shammari A., Alwahibi M., Soliman D., Dewir Y.H. (2020). Linalool-loaded glutathione-modified gold nanoparticles conjugated with CALNN peptide as apoptosis inducer and NF-κB translocation inhibitor in SKOV-3 cell line. Int. J. Nanomed..

[33] Zhu X., Zeng X., Zhang X., Cao W., Wang Y., Chen H., Wang T., Tsai H.-I., Zhang R., Chang D. (2016). The effects of quercetin-loaded PLGA-TPGS nanoparticles on ultraviolet B-induced skin damages in vivo. Nanomed. Nanotechnol. Biol. Med..

[34] Lu H., Chen X., Xu H. (2021). Preparation of Galangin Self-microemulsion Drug Delivery System and Evaluation of Its Pharmacokinetics In Vivo and Antioxidant Activity In Vitro. Preprints.

[35] Jullian C. (2009). Improvement of galangin solubility using native and derivative cyclodextrins: An UV-Vis and NMR study. J. Chil. Chem. Soc..

[36] Jeong D., Jeong J.P., Dindulkar S.D., Cho E., Yang Y.H., Jung S. (2016). Cyclosophoraose/cellulose hydrogels as an efficient delivery system for galangin, a hydrophobic antibacterial drug. Cellulose.

[37] Yao H., Lu H., Zhang J., Xue X., Yin C., Hu J., Zou R., Wang L., Xu H. (2019). Preparation of prolonged-circulating galangin-loaded liposomes and evaluation of antitumor efficacy in vitro and pharmacokinetics in vivo. J. Nanomater..

[38] Matshetshe K.I., Parani S., Manki S.M., Oluwafemi O.S. (2018). Preparation, characterization and in vitro release study of β-cyclodextrin/chitosan nanoparticles loaded Cinnamomum zeylanicum essential oil. Int. J. Biol. Macromol..

[39] Mignet N., Seguin J., Chabot G.G. (2013). Bioavailability of polyphenol liposomes: A challenge ahead. Pharmaceutics.

[40] Sabry S., Okda T., Hasan A. (2021). Formulation, characterization, and evaluation of the anti-tumor activity of nanosized galangin loaded niosomes on chemically induced hepatocellular carcinoma in rats. J. Drug Deliv. Sci. Technol..

[41] Davila Y.A., Sancho M.I., Almandoz M.C., Gasull E. (2018). Spectroscopic and electronic analysis of chelation reactions of galangin and related flavonoids with nickel (II). J. Chem. Eng. Data.

[42] Jingou J., Shilei H., Weiqi L., Danjun W., Tengfei W., Yi X. (2011). Preparation, characterization of hydrophilic and hydrophobic drug in combine loaded chitosan/cyclodextrin nanoparticles and in vitro release study. Colloids Surf. B Biointerfaces.

[43] Sahu S.K., Mallick S.K., Santra S., Maiti T.K., Ghosh S.K., Pramanik P. (2010). In vitro evaluation of folic acid modified carboxymethyl chitosan nanoparticles loaded with doxorubicin for targeted delivery. J. Mater. Sci. Mater. Med..

[44] Barone G., Bartoli L., Belfiore C.M., Crupi V., Longo F., Majolino D., Mazzoleni P., Venuti V. (2011). Comparison between TOF-ND and XRD quantitative phase analysis of ancient potteries. J. Anal. At. Spectrom..

[45] Gao Y.A., Li Z.H., Du J.M., Han B.X., Li G.Z., Hou W.G., Shen D., Zheng L.-Q., Zhang G.Y. (2005). Preparation and characterization of inclusion complexes of β-cyclodextrin with ionic liquid. Chem. Eur. J..

[46] Honary S., Jahanshahi M., Golbayani P., Ebrahimi P., Ghajar K. (2010). Doxorubicin-loaded albumin nanoparticles: Formulation and characterization. J. Nanosci. Nanotechnol..

[47] Fornaguera C., Solans C. (2018). Characterization of polymeric nanoparticle dispersions for biomedical applications: Size, surface charge and stability. Pharm. Nanotechnol..

[48] Suvarna S., Das U., Kc S., Mishra S., Sudarshan M., Saha K.D., Narayana Y. (2017). Synthesis of a novel glucose capped gold nanoparticle as a better theranostic candidate. PLoS ONE.

[49] Balakrishnan S., Mukherjee S., Das S., Bhat F.A., Raja Singh P., Patra C.R., Arunakaran J. (2017). Gold nanoparticles–conjugated quercetin induces apoptosis via inhibition of EGFR/PI3K/Akt–mediated pathway in breast cancer cell lines (MCF-7 and MDA-MB-231). Cell Biochem. Funct..

[50] Danaei M., Dehghankhold M., Ataei S., Hasanzadeh Davarani F., Javanmard R., Dokhani A., Khorasani S., Mozafari M.R. (2018). Impact of particle size and polydispersity index on the clinical applications of lipidic nanocarrier systems. Pharmaceutics.

[51] Seethalakshmi S., Suja S. (2020). Biosynthesis of nanocomposite from Alpinia galanga for phytochemical and characterisation analysis for orthopaedic replacements. J. Univ. Shanghai Sci. Technol..

[52] Mühlfeld C., Rothen-Rutishauser B., Vanhecke D., Blank F., Gehr P., Ochs M. (2007). Visualization and quantitative analysis of nanoparticles in the respiratory tract by transmission electron microscopy. Part. Fibre Toxicol..

[53] Lu Y., Wang L., Chen D., Wang G. (2012). Determination of the concentration and the average number of gold atoms in a gold nanoparticle by osmotic pressure. Langmuir.

[54] Junthip J., Tabary N., Leclercq L., Martel B. (2015). Cationic β-cyclodextrin polymer applied to a dual cyclodextrin polyelectrolyte multilayer system. Carbohydr. Polym..

[55] Scott M.D., Van den Berg J.J., Repka T., Rouyer-Fessard P., Hebbel R.P., Beuzard Y., Lubin B.H. (1993). Effect of excess alpha-hemoglobin chains on cellular and membrane oxidation in model beta-thalassemic erythrocytes. J. Clin. Investig..

[56] Chen Y., Deuster P. (2009). Comparison of quercetin and dihydroquercetin: Antioxidant-independent actions on erythrocyte and platelet membrane. Chem.-Biol. Interact..

[57] Ferrali M., Signorini C., Caciotti B., Sugherini L., Ciccoli L., Giachetti D., Comporti M. (1997). Protection against oxidative damage of erythrocyte membrane by the flavonoid quercetin and its relation to iron chelating activity. FEBS Lett..

[58] Mazzarino L., Loch-Neckel G., dos Santos Bubniak L., Ourique F., Otsuka I., Halila S., Pedrosa R.C., Santos-Silva M.C., Lemos-Senna E., Muniz E.C. (2015). Nanoparticles made from xyloglucan-block-polycaprolactone copolymers: Safety assessment for drug delivery. Toxicol. Sci..

[59] Choi J., Reipa V., Hitchins V.M., Goering P.L., Malinauskas R.A. (2011). Physicochemical characterization and in vitro hemolysis evaluation of silver nanoparticles. Toxicol. Sci..

[60] Mosmann T. (1983). Rapid colorimetric assay for cellular growth and survival: Application to proliferation and cytotoxicity assays. J. Immunol. Methods.

[61] Zhong Z., Chen X., Tan W., Xu Z., Zhou K., Wu T., Wang Y. (2011). Germacrone inhibits the proliferation of breast cancer cell lines by inducing cell cycle arrest and promoting apoptosis. Eur. J. Pharmacol..

[62] Liu D., You P., Luo Y., Yang M., Liu Y. (2018). Galangin induces apoptosis in MCF-7 human breast cancer cells through mitochondrial pathway and phosphatidylinositol 3-kinase/Akt inhibition. Pharmacology.

[63] Gholam-Hosseinpour M., Karami Z., Hamedi S., Mehri Lighvan Z., Heydari A. (2022). Enhancing in vitro cytotoxicity of doxorubicin against MCF-7 breast cancer cells in the presence of water-soluble β-cyclodextrin polymer as a nanocarrier agent. Polym. Bull..

[64] Śliwka L., Wiktorska K., Suchocki P., Milczarek M., Mielczarek S., Lubelska K., Flis A. (2016). The comparison of MTT and CVS assays for the assessment of anticancer agent interactions. PLoS ONE.

[65] Berardi A., Bisharat L. (2014). Nanotechnology systems for oral drug delivery: Challenges and opportunities. Nanotechnol. Drug Deliv..

[66] Gavas S., Quazi S., Karpiński T.M. (2021). Nanoparticles for Cancer Therapy: Current Progress and Challenges. Nanoscale Res. Lett..

[67] Ali S.H., Sulaiman G.M., Al-Halbosiy M.M.F., Jabir M.S., Hameed A.H. (2019). Fabrication of hesperidin nanoparticles loaded by poly lactic co-Glycolic acid for improved therapeutic efficiency and cytotoxicity. Artif. Cells Nanomed. Biotechnol..

[68] Natarajan T., Anandhi M., Aiswarya D., Ramkumar R., Kumar S., Perumal P. (2016). Idaein chloride induced p53 dependent apoptosis in cervical cancer cells through inhibition of viral oncoproteins. Biochimie.

[69] Ha T.K., Kim M.E., Yoon J.H., Bae S.J., Yeom J., Lee J.S. (2013). Galangin induces human colon cancer cell death via the mitochondrial dysfunction and caspase-dependent pathway. Exp. Biol. Med..

[70] Redza-Dutordoir M., Averill-Bates D.A. (2016). Activation of apoptosis signalling pathways by reactive oxygen species. Biochim. Biophys. Acta (BBA)-Mol. Cell Res..

[71] Wang H.X., Tang C. (2017). Galangin suppresses human laryngeal carcinoma via modulation of caspase-3 and AKT signaling pathways. Oncol. Rep..

[72] Mangla B., Neupane Y.R., Singh A., Kumar P., Shafi S., Kohli K. (2020). Lipid-nanopotentiated combinatorial delivery of tamoxifen and sulforaphane: Ex vivo, in vivo and toxicity studies. Nanomedicine.

[73] Yang D.K., Kang H.S. (2018). Anti-diabetic effect of cotreatment with quercetin and resveratrol in streptozotocin-induced diabetic rats. Biomol. Ther..

[74] Rajeh M.A.B., Kwan Y.P., Zakaria Z., Latha L.Y., Jothy S.L., Sasidharan S. (2012). Acute toxicity impacts of Euphorbia hirta L extract on behavior, organs body weight index and histopathology of organs of the mice and Artemia salina. Pharmacogn. Res..

[75] Jasper R., Locatelli G.O., Pilati C., Locatelli C. (2012). Evaluation of biochemical, hematological and oxidative parameters in mice exposed to the herbicide glyphosate-Roundup^®^. Interdiscip. Toxicol..

[76] Chen Z., Meng H., Xing G., Chen C., Zhao Y., Jia G., Wang T., Yuan H., Ye C., Zhao F. (2006). Acute toxicological effects of copper nanoparticles in vivo. Toxicol. Lett..

[77] Chen H., Dorrigan A., Saad S., Hare D.J., Cortie M.B., Valenzuela S.M. (2013). In vivo study of spherical gold nanoparticles: Inflammatory effects and distribution in mice. PLoS ONE.

[78] Tsai M.S., Chien C.C., Lin T.H., Liu C.C., Liu R.H., Su H.L., Chiu Y.-T., Wang S.H. (2015). Galangin prevents acute hepatorenal toxicity in novel propacetamol-induced acetaminophen-overdosed mice. J. Med. Food.

